# Particle fractionation controls *Escherichia coli* release from solid manure

**DOI:** 10.1016/j.heliyon.2021.e07038

**Published:** 2021-05-25

**Authors:** Nasrollah Sepehrnia, Sayyed-Hassan Tabatabaei, Hamdollah Norouzi, Mohsen Gorakifard, Hossein Shirani, Fereidoun Rezanezhad

**Affiliations:** a Institute of Soil Science, Leibniz Universität Hannover, Herrenhäuser Str. 2, D-30419 Hannover, Germany; b Department of Water Engineering, Faculty of Agriculture, Shahrekord University, Shahrekord, Iran; c Department of Mechanical Engineering, Universitat Rovira i Virgili, Spain; d Department of Soil Science, Faculty of Agriculture, Vali-e-Asr University of Rafsanjan, Rafsanjan, Iran; e Ecohydrology Research Group, Department of Earth and Environmental Sciences and Water Institute, University of Waterloo, Waterloo, Canada

**Keywords:** Manure particle size, Solid manure, Release curve, Bacteria, Manure management, Breakthrough curve

## Abstract

Bacteria transport through soil is a complex process particularly when the cells are released from solid manures and co-transported with particles. This study focuses on understanding of the *Escherichia coli* release from different particle fractions (0.25-, 0.5-, 1-, and 2-mm) of solid manure and evaluating different influent boundary conditions during cell release from manure and when a solid manure is applied to the soil. The 0.25-mm and 2-mm particle sizes resulted a greater cell release compared to 0.5-mm and 1-mm fractions (*p* < 0.05). The shape and magnitude of the cell release curves (CRCs) from the original manure bulk were mainly influenced by the two 0.25-mm and 2-mm fractions, respectively. The arithmetic mean for normalizing the CRCs and the time variable- based normalized CRCs for the manure-treated soil were the robust variables in evaluation of the experimental data. However, a single maximum bacteria concentration could provide the realistic dataset for the modeling process. Evaluation of the root-mean-squared-error and Akaike criterion showed that the two- and three-parametric models are recommended for simulating the cell release from solid manure in comparison with one parametric models. This study also suggests considering separate microbial release evaluations, with regards to influent concentration, for manure and manure-treated soils to propose best management practices for controlling bacteria pollution. Further research will reveal the key roles of different woody components and soluble material ratios for the various solid manures in bacteria release.

## 1 Introduction

Considerable manure is produced from cattle farms and applied to agricultural fields (ASAE Standards, 2005; Fangueiro et al., 2014, Koneswaran and Nierenberg, 2008). The distributed manures are a principle source of bacterial pollution as bacteria cells are released by rainfall and irrigation events and enter surface and ground water (Blaustein et al., 2015; Font-Palma, 2019; Manyi-Loh et al., 2016). The release of bacteria from manures and transport into water resources can adversely affect water quality and human health (Bradford et al., 2013; Hong et al., 2018; Mantha et al., 2017; Sepehrnia et al., 2019).

Several management practices have been proposed in international protocols for solid and liquid wastes to be chemically, physically, and biologically treated (UNECE, 1999; UN, 1997; Wright, 2005). The main processes are predominantly used in aerobic treatment by composting and anaerobic digestion, as well as drying a large part of manures in or out of confined facilities (Loyon, 2018; NRC, 2002). Composting, stacking, and drying change and reduce the manure particle size and the number of bacteria (ASAE Standards, 2005). Notwithstanding, the standard and most common management practice for manures, whether raw or dried, is land application without any necessary pathogen treatment in contrast to sewage sludge application (*i.e.,* so-called “biosolids”), which typically require pretreatment (Loyon et al., 2016; Loyon, 2018). This, however, provides routes by which pathogens can contaminate natural environments, food crops, animals, and humans (Bradford et al., 2006; Glæsner et al., 2015; Muirhead et al., 2005).

Animal manures are classified as either liquid or solid depending on consistency of solid materials (Blaustein et al., 2015). In general, manures are considered solid with a dry solid content above 20% (Blaustein et al., 2015). Woody, soluble, and colloidal materials with a broad size and distribution mainly constitute solid manures (Hafez et al., 1974; Sepehrnia et al., 2017, 2018). The composition depends on type, age, and nutrition of animal, bedding materials, carbon compounds, storage conditions, etc. that can naturally affect bacteria counts in the manure (Blaustein et al., 2015; Unc and Goss, 2004).

Studies have shown that manures commonly include about 10^10^ bacteria g^−1^ dry manure, and pathogenic forms, if present can be nearly 10^5^ colony forming units (CFU) g^−1^ (*e.g.,* Unc and Goss, 2004). However, accurately estimating and determining the initial bacteria concentration in prepared suspensions and/or liquid manures (*i.e.,* slurry) is still a big challenge in bacteria transport studies as a precondition to estimating and managing microbial water quality (Guber et al., 2005; 2007a,b, 2013). This will be even more problematic if the solid manures are considered a source of pollution because heterogeneities in such complex media influence cell release from solid bulk and govern cell transport, retention, and release through soil (Blaustein et al., 2015, 2016; Sepehrnia et al., 2017).

The proper evaluation of bacteria release and transport from manures, with respect to heterogeneous composition and as the harbor for bacterial communities is thus a necessity for manure management. A two-stage process with a fast initial release followed by a slower log−linear release has been proposed for the release kinetics of protozoa oocysts from solid animal waste under single-dripper raindrops (Bradford and Schijven, 2002; Schijven et al., 2004). Blaustein et al. (2015, 2016) determined the effects of rainfall intensity and surface slope on the release of *Escherichia coli* (*E. coli*) from solid dairy manure to assess the performance of the one-parametric exponential model and the two-parametric Bradford−Schijven model (Bradford and Schijven, 2002). The bacterial release occurred in two stages that corresponded to mechanisms associated with release of manure liquid- and solid-phases and recommended the Bradford−Schijven model for simulating bacterial release from solid manure. There is little information available to address the way bacteria are released from different fractions of solid manures having complex pore space, before they access soil medium, and literatures have indicated substantial gaps in our understanding in this regards (Blaustein, 2014; Blaustein et al., 2015, 2016; Stocker et al., 2020a, 2020b), so that the models have been mostly focused and tested on slurries (Bradford and Schijven, 2002; Schijven et al., 2004).

In this study, we assessed the release of *E. coli* from four different fractions (*i.e.,* 0.25-, 0.5-, 1-, and 2-mm) of an air-dried fresh solid cow (*Bos taurus*) manure. We hypothesized that the individual fractions of a solid cow manure could vary in composition, pore space, and *E. coli* concentration, which resulted in different trends of cell release. The main objectives of this study were to: (i) compare *E. coli* release from different fractions of solid cow manure; (ii) evaluate four available release models of different fractions of solid cow manure; (iii) find the best criterion for the initial influent concentration(s), *C*_*0*_, to normalize manure release and soil breakthrough curves to have unique data evaluation. With this, knowledge of bacteria release from manures and transport, retention, and release through soil facilitate manure management in field.

## 2 Material and methods

### 2.1 Manure sample preparation and properties

Fresh cow manure was collected as excreted (less than 5 min after deposition) and the manure was air-dried in shade for 72 h at room temperature. Dried materials were passed through four different sieve sizes of 0.25-, 0.5-, 1-, and 2-mm in separate operations. These fractions preserved the original manure particle size distribution and fraction heterogeneity as the particle size increased. Approximately 5 g (equal to 30 Mg ha^−1^, dry basis) of each manure fraction were used for leaching experiments, where the water content of the air-dried manure fraction was considered (see Table 1). The manure fractions were kept at 4 °C for the leaching experiment and further analysis.

**Table 1:** Initial *Escherichia coli* concentration and water content for different manure fractions.

Manure	≤0.25-mm	≤0.5-mm	≤1-mm	≤2-mm	C_fresh_	average
*E. coli* (CFU mL^−1^)	14.00×10^6^ (±0.61×10^6^)	7. 05×10^6^ (±0.62×10^6^)	8.50×10^6^ (±0.37×10^6^)	12.00×10^6^ (±0.47×10^6^)	7.00×10^6^ (±3. 5×10^6^)	10. 40×10^6^ (±0.47×10^6^)
*θ*_*m*_ (%)	38.00 (±4.0)^a^	36.00 (±2.0)^b^	36.00 (±2.0)^b^	29.00 (±2.0)^c^	90.00 (±3.0)	‒

### 2.2 Leaching experiment

A series of leaching experiments were performed for the manure with different fractions based on the saturated pore volume (i.e., PV; PV = θ_v_×V_t_) of a sandy soil (83.40% sand, 6.72% silt, 9.88% clay, and 0.22% organic matter). The bulk of the manure fraction was uniformly spread on the top plate of a funnel, then, ten times 6.6 ml (0.1 PV) and nineteen times 66 ml (1 PV) tap water for the first and subsequent 19 PVs were continuously poured, at a constant rate, on the manure fraction. Such fine (0.1 PV) and moderate (1 PV) increments in effluent sampling provided a better trend for cell release during leaching. The manure fractions were leached up to 20 PVs. The effluents were sampled at times equivalent to the leaching increments. Therefore, 29 samples were collected using sterile containers for each manure fraction.

### 2.3 Cell recovery

The effluent bacteria from the leaching experiment were recovered by plate-count method (Swanson et al., 1992; Guber et al., 2006) according to previous studies by Sepehrnia et al. (2017, 2018). One mL of the effluents was poured into 9 ml sterilized distilled water, diluted (*e.g.,*1,000 and 10,000 times), and 0.1 ml of diluted sample plated on Eosin Methylene Blue (EMB) and incubated at 37 °C for 18–24 h. The viable cells grown (*i.e.,* greenish metallic sheen) on the medium culture plates were counted and the concentrations of bacteria were reported based on the colony forming units (CFUs) of fecal coliforms per 1 ml (CFU mL^−1^) (Swanson et al., 1992; Guber et al., 2006).

### 2.4 Release modeling

The concentrations of *E. coli* in the effluents released from manure were converted to cumulative total numbers of bacteria (based on flow rate) that were removed with each effluent of manure fraction. The cumulative amounts of released cells in the leachate were interpolated to determine the total time-dependent release from manure. The release of *E. coli* for each manure fraction were modeled as a function of time with Eq. (1); the one-parametric exponential dependence; Eq. (2); the two-parametric Bradford and Schijven (2002), Eq. (3); Vadas et al. (2004), and Eq. (4); the three-parametric Guber et al. (2013) model as follows:

(1) $$\frac{N}{N_{0}}=1-e^{\left(-k_{e}W\right)}$$

(2) NN0=1−1(1+kpβW)1β

(3) $$\frac{N}{N_{0}}=A{k_{p}}^{\beta }$$

(4) NN0=Er(1−1(1+kpβW)1β)

where N [CFU m^−2^] is the total number of bacteria per unit area of manure application [m^−2^]; N_0_ [CFU m^−2^] is the initial number of bacteria per unit area of manure application; W [mm] is rainfall depth; *k*_*e*_ [mm^−1^] and *k*_*p*_ [mm^−1^] are constants; *β* [-] is a dimensionless shape parameter; A [cm^−1^] is the rate constant parameter; *Er* [-] is the parameter for release efficiency. Note that we adapted and unified the characters of the equations to simplify model comparisons. For example, *β* as the dimensionless shape parameter in Eq. (3) is shown with n in Vadas et al. (2004). The original forms of the given equations can be found in Blaustein (2014). All equations were fitted to the “time-release” data with a FORTRAN code REL_BACT developed by Blaustein (2014), which was based on the Marquardt-Levenberg optimization algorithm as implemented by van Genuchten (1981).

### 2.5 Data analyses

The data analyses were separated into two parts; the cell release curves (CRCs, plotted in CFU ml^−1^ vs. PV), and the modeling. First, nine PVs were randomly selected using MATLAB and the concentrations of released bacteria from the four fractions were compared in a full factorial statistical design in SPSS (IBM statics; version 26). Second, the optimized model parameters were compared for the four boundary conditions of the *C*_*0*_ with regard to the manure fraction size. The results for the first and the second evaluations are presented in Tables 2 and 3a and 3b, respectively. The performances of the release models were assessed using root-mean-squared-error (RMSE) and Akaike information criterion (AIC) values. The AIC index included in the REL_BACT code considered the number of fitting parameters (*i.e.,* one, two, and three) given in Eqs. (1), (2), (3), and (4). RMSE and the corrected AIC were computed in REL_BACT code using Eqs. (5) and (6), respectively.

(5) $$\text{RMSE}=\sqrt{\frac{\text{RSS}}{n}}$$

(6) $$\text{AIC}=\text{nln}\left(\frac{\text{RSS}}{n}\right)+2k+\frac{2k\left(k+1\right)}{n-k-1}$$

where RSS is the residual sum of squares, *n* and *k* are number of measurements and model parameters, respectively (both RMSE and AIC units are dimensionless). It should be noted that a smaller RMSE and a more negative corrected AIC values indicate better model performance.

**Table 2:** Mean *Escherichia coli* concentration (×10^6^ CFU ml^−1^) released from four manure fractions in nine randomly selected pore volumes. STD means standard deviation. Values in the parentheses are standard deviation of triplicate. Average refers to the arithmetic average of cells found in the given fractions.

*E. coli*	0.25-mm	0.5-mm	1-mm	2-mm
**C**_**0.3PV**_	2.00 (±3.61)	2.90 (±5.57)	1.00 (±2.65)	2.30 (±4.58)
**C**_**0.7PV**_	4.93 (±7.10)	4.93 (±7.09)	1.17 (±2.52)	2.70 (±3.61)
**C**_**0.9PV**_	7.13 (±4.20)	3.70 (±7.02)	1.10 (±3.00)	4.20 (±4.58)
**C**_**5PV**_	2.1 (±4.04)	4.00 (±6.56)	2.37 (±5.51)	3.73 (±7.51)
**C**_**7PV**_	0.97 (±2.52)	2.23 (±3.06)	1.13 (±2.1)	1.47 (±6.51)
**C**_**8PV**_	0.770 (±1.52)	2.57 (±3.51)	0.93 (±2.31)	1.30 (±5.29)
**C**_**13PV**_	0.040 (±0.10)	0.28 (±0.15)	0.23 (±0.21)	0.087 (±0.35)
**C**_**17PV**_	0.043 (±0.15)	0.15 (±0.35)	3.57 (±0.86)	0.12 (±0.36)
**C**_**18PV**_	0.003 (±0.06)	0.077 (±0.12)	1.93 (±0.51)	0.23 (±0.45)
**Average**	1.99 (±2.4)	2.27 (1.74)	1.79 (±1.53)	1.75 (1.76)

**Table 3a:** Average and standard deviation of parameters from the exponential, the Bradford and Schijven (2002), Vadas et al. (2004), and Guber et al. (2013) models fitted to the effluent data under different manure fraction and boundary conditions (i.e., average and max values).

*C*_*0*_ = average	model; parameter; units	≤0.25-mm	≤0.5-mm	≤1-mm	≤2-mm
Exponential	Eq. (1); K_e_; mm^−1^	0.1192 (±0.002)	0.1563 (±0.0182)	0.1380 (±.0214)	0.0772 (±0.007)
Bradford and Schijven (2002)	Eq. (2); K_p_; mm^−1^	–	–	–	0.00001 (±0.00002)
	Eq. (2); *β*; dimensionless	–	–	–	0.1695 (±0.0403)
Vadas et al. (2004)	Eq. (3); K_p_; mm^−1^	0.7261 (±0.0146)	0.6242 (±0.08)	0.5079 (±0.0616)	0.3631 (±.0279)
	Eq. (3); *β*; dimensionless	0.3285 (±0.0031)	0.3553 (±0.0166)	0.3418 (±0.0231)	0.3769 (±0.0196)
Guber et al. (2013)	Eq. (4); K_p_; mm^−1^	0.0009 (±0.0004)	0.0188 (±0.0051)	0.0196 (±0.0037)	0.0087 (±0.0014)
	Eq. (4); *β*; dimensionless	0.4454 (±0.0351)	1.0575 (±0.0757)	1.0283 (±0.0561)	0.8326 (±0.0101)
	Eq. (4); Er; dimensionless	5.2390 (±0.1817)	6.3465 (±0.1696)	4.0245 (±0.0346)	3.9537 (0.2297)
*C*_*0*_ = max	model; parameter; units	≤0.25-mm	≤0.5-mm	≤1-mm	≤2-mm
Exponential eq	Eq. (1); K_e_; mm^−1^	0.0038 (±0.0004)	0.0035 (±0.0003)	0.0014 (±.0001)	0.0015 (±0.0001)
Bradford and Schijven (2002)	Eq. (2); K_p_; mm^−1^	0.0234 (±0.0011)	0.0219 (±0.0033)	0.0212 (±0.0014)	0.0157 (±0.0015)
	Eq. (2); *β*; dimensionless	1.3720 (±0.0594)	1.3817 (±0.0381)	2.0733 (±0.1446)	1.7410 (±0.1238)
Vadas et al. (2004)	Eq. (3); K_p_; mm^−1^	0.0944 (±0.0019)	0.0812 (±0.0104)	0.0620 (±0.0056)	0.0472 (±0.0036)
	Eq. (3); *β*; dimensionless	0.3285 (±0.0031)	0.3553 (±0.0166)	0.3224 (±0.0167)	0.3769 (±0.0195)
Guber et al. (2013)	Eq. (4); K_p_; mm^−1^	0.0010 (±0.0006)	0.0185 (±0.0036)	0.0215 (±.0042)	0.0087 (±0.0014)
	Eq. (4); *β*; dimensionless	0.4454 (±0.0352)	1.0783 (±0.0655)	0.5296 (±0.5104)	0.8326 (±0.0100)
	Eq. (4); Er; dimensionless	0.6811 (±0.0236)	0.8298 (±0.0171)	0.5075 (±0.0273)	0.5139 (±0.0299)

**Table 3b:** Average and standard deviation of parameters from the exponential, the Bradford and Schijven (2002), Vadas et al. (2004), and Guber et al. (2013) models fitted to the effluent data under different manure fraction and boundary conditions (i.e., 0.5-mm and Time-variable max values).

*C*_*0*_ = 0.5-mm	model; parameter; units	≤0.25-mm	≤0.5-mm	≤1-mm	≤2-mm
Exponential	Eq. (1); K_e_; mm^−1^	0.234 (±0.003)	0.424 (±0.189)	0.396 (±0.067)	0.410 (±0.044)
Bradford and Schijven (2002)	Eq. (2); K_p_; mm^−1^	–	–	–	–
	Eq. (2); *β*; dimensionless	–	–	–	–
Vadas et al. (2004)	Eq. (3); K_p_; mm^−1^	2.178 (±0.061)	2.849 (±3.921)	0.873 (±0.036)	3.333 (±0.256)
	Eq. (3); *β;* dimensionless	0.392 (±0.073)	0.448 (±0.114)	0.375 (±0.149)	0.377 (±0.020)
Guber et al. (2013)	Eq. (4); K_p_; mm^−1^	0.003 (±0.001)	0.0004 (±0.0002)	0.0003 (±0.0002)	0.009 (±0.001)
	Eq. (4); *β*; dimensionless	0.594 (±0.055)	1.963 (±0.027)	2.126 (±0.053)	0.833 (±0.010)
	Eq. (4); Er; dimensionless	18.900 (±0.847)	1542.500 (±730.441)	1642.100 (±2012.0)	36.277 (±2.110)
*C*_*0*_ = Time-var max	Model; parameter; units	≤0.25-mm	≤0.5-mm	≤1-mm	≤2-mm
Exponential	Eq. (1); K_e_; mm^−1^	0.210 (±0.003)	0.228 (±0.029)	0.267 (±0.038)	0.170 (±0.017)
Bradford and Schijven (2002)	Eq. (2); K_p_; mm^−1^	–	–	0.00001	–
	Eq. (2); *β*; dimensionless	–	–	0.137	–
Vadas et al. (2004)	Eq. (3); K_p_; mm^−1^	1.386 (±0.032)	1.020 (±0.569)	0.609 (±0.022)	0.804 (±0.062)
	Eq. (3); *β*; dimensionless	0.319 (±0.054)	0.436 (±0.135)	0.458 (±0.012)	0.377 (±0.020)
Guber et al. (2013)	Eq. (4); K_p_; mm^−1^	0.009 (±0.001)	0.006 (±0.005)	0.001 (±0.002)	0.009 (±0.001)
	Eq. (4); *β*; dimensionless	0.761 (±0.052)	1.112 (±0.655)	2.120 (±0.115)	0.832 (±0.010)
	Eq. (4); Er; dimensionless	12.090 (±0.781)	452.093 (±762.888)	631.420 (±475.735)	8.746 (±0.510)

## 3 Results

### 3.1 Manure fractions and bacteria release

The average concentration of bacteria for bulk manure fractions and fresh manure are shown in Table 1. There were slightly more cells in the 0.25-mm and 2-mm bulk fractions ranging from .7.5 × 10^6^–14.0 × 10^6^ CFU ml^−1^. The concentration of bacteria in different fractions was not significantly affected by drying if compared with fresh origin manure. The water content of manure fractions were different (*p* < 0.05, Table 1). Font-Palma (2019) reviewed different cattle manure properties and they reported water content from Shen et al. (2015) and Wang et al. (2011) studies for the fresh cattle and beef cattle manures about 70.7% and 75.66%, respectively, which were comparable with our measurement for fresh cow manure (Table 1). The concentrations of *E. coli* measured for the manure fractions were also corresponded with the previous studies in which the either fresh or the (shadow) dried solid manures reported (Blaustein et al., 2015; Guber et al., 2013; Sepehrnia et al., 2014, 2017, 2018; Unc and Goss, 2004). Unc and Goss (2004) and Kabelitza et al. (2020) also reported that cultivable bacteria are commonly about 10^9^–10^10^ CFU g^−1^ in fresh solid manures (i.e., cow, poultry, pig). Blaustein et al. (2015) observed *E. coli* constituted over half of total coliforms, and the total contents of *E. coli* and *enterococci* in the synthesized solid manure were similar (2.62 ± 0.76 × 10^6^ CFU ml^−1^). Guber et al. (2013) reported 0.76–2.67 × 10^6^ CFU ml^−1^ for *E. coli* released from cow manure.

**Figure 1:**
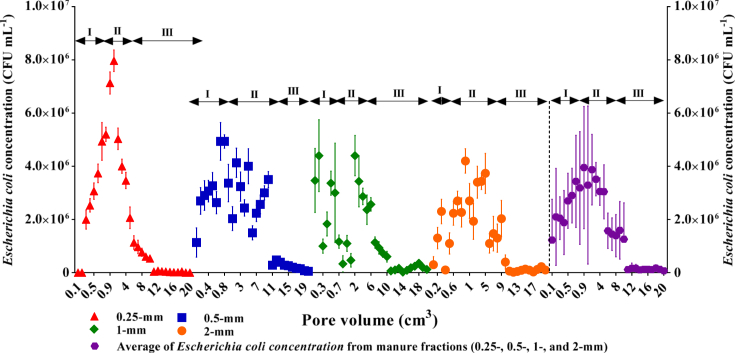
*Escherichia coli* release curves from different manure fractions after 20 pore volumes leaching. Release phases (I, II, III) are illustrated for each fraction and average of bacteria from the studied fractions (0.25-, 0.5-, 1-, and 2-mm). The bars represent standard deviation.

The cell release curves (CRCs, CFU ml^−1^) are shown in Figure 1. The trend for the increase of release was observed up to 2 PVs (phase I) and then began decreasing up to 11 PVs (phase II) and finally the release entered a stationary mode (phase III) (see Figure 1). The effect of manure fractions was distinguished at 1 to 6 PVs where the 0.5-mm, 1-mm, and 2-mm fractions showed similar decrease in cell release, while the 0.25-mm had the highest concentration (Table 1). For this observation, the evidence was the higher slope in decreasing mode and/or the higher number of total cells observed for the 0.25-mm fraction in the nine randomly selected PVs as shown in Table 2, particularly when compared with the 1- and 2-mm manure fractions. The latter was investigated by multiple comparisons where the effects of the fraction size, PV, and the interaction of size×PV were significantly different with respect to bacteria release (*p* < 0.001). The mean concentrations of bacteria are presented in Table 2.

### 3.2 Initial concentrations to normalize release curves

**Figure 2:**
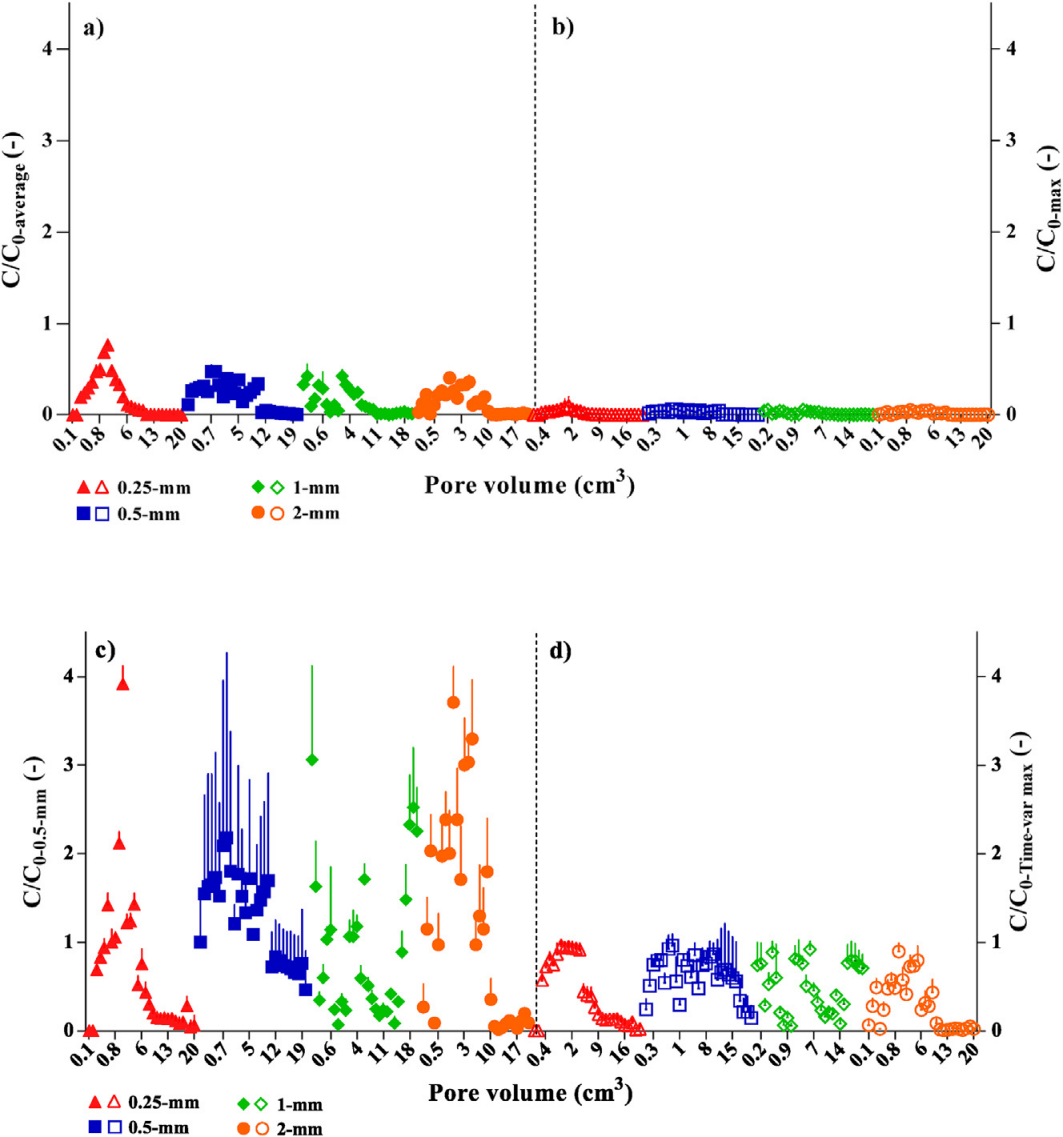
The release bacteria curves from four different manure fractions normalized using the: (a) average, (b) maximum and the time variable concentration conditions; (c) the 0.5-mm concentration values, and (d) maximum concentration values selected among all fractions at each pore volume, as the reference concentrations *C*_*0*_*,* during the leaching period. The bars represent standard deviation.

**Figure 3:**
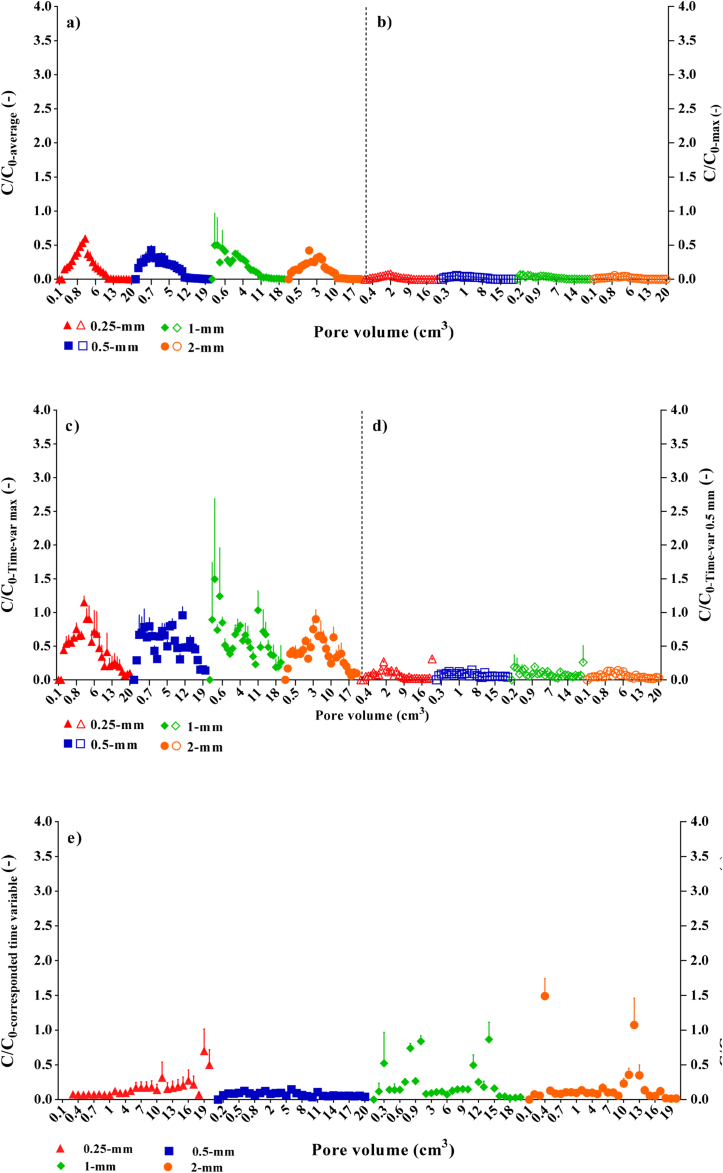
The breakthrough curves of bacteria from different manure fractions amended with a sandy soil. The curves normalized when a single concentration; (a) average and (b) maximum values, and time variable concentration conditions; (c) time variable max values (Time-var max) selected among all fractions at each pore volume (PV), (d) time variable values of the 0.5-mm fraction (Time-var 0.5-mm), and (e) corresponded time variable concentration conditions (*e.g., C*/*C*_*0*_ resulted from 1 PV manure-amended soil *C*, divided by corresponded 1 PV manure fraction *C*_*0*_) are considered as the *C*_*0*_. The bars represent standard deviation.

The average value of bacteria cells (10.4×10^6^ ± 0.520 × 10^6^ CFU ml^−1^), for all manure fractions (Table 1), was considered as the *C*_*0*_ for normalized concentrations (*i.e., C*/*C*_*0*_) in the CRCs (Figure 2a). Two other considerations were a single maximum concentration (max values) observed during the leaching among the manure fractions (80.00 × 10^6^ CFU ml^−1^, Figure 2b) and the time variable concentration, respectively. The latter consisted of three datasets: a) the time variable concentrations of the fraction in which the greatest frequency of the highest concentrations was observed if all concentrations of the manure fractions were compared and considered for a PV (in this case, the 0.5-mm fraction was selected, Figure 2c; *i.e.,* 0.5-mm fraction (it had 14 maximum measured data out of 29); b) the time variable concentrations made by selecting the maximum values of each PV (Time-var max), when different manure fractions were compared (Figure 2d); c), the time variable concentration from the independent manure leaching for each fraction size may be applied to soil (*e.g., C* at 0.25 PV from soil vs. *C_0_* at 0.25 PV from manure) (Figure 3). The last hypothesis of the time variable boundary condition was to find if only one dataset was enough to make soil breakthrough curves (BTCs) and cell release curves from manure or if separate evaluation was needed. All the initial boundary conditions are separately discussed in the following. The data are mean values of three replicates for each PV.

### 3.3 Modeling kinetic cell release

**Figure 4:**
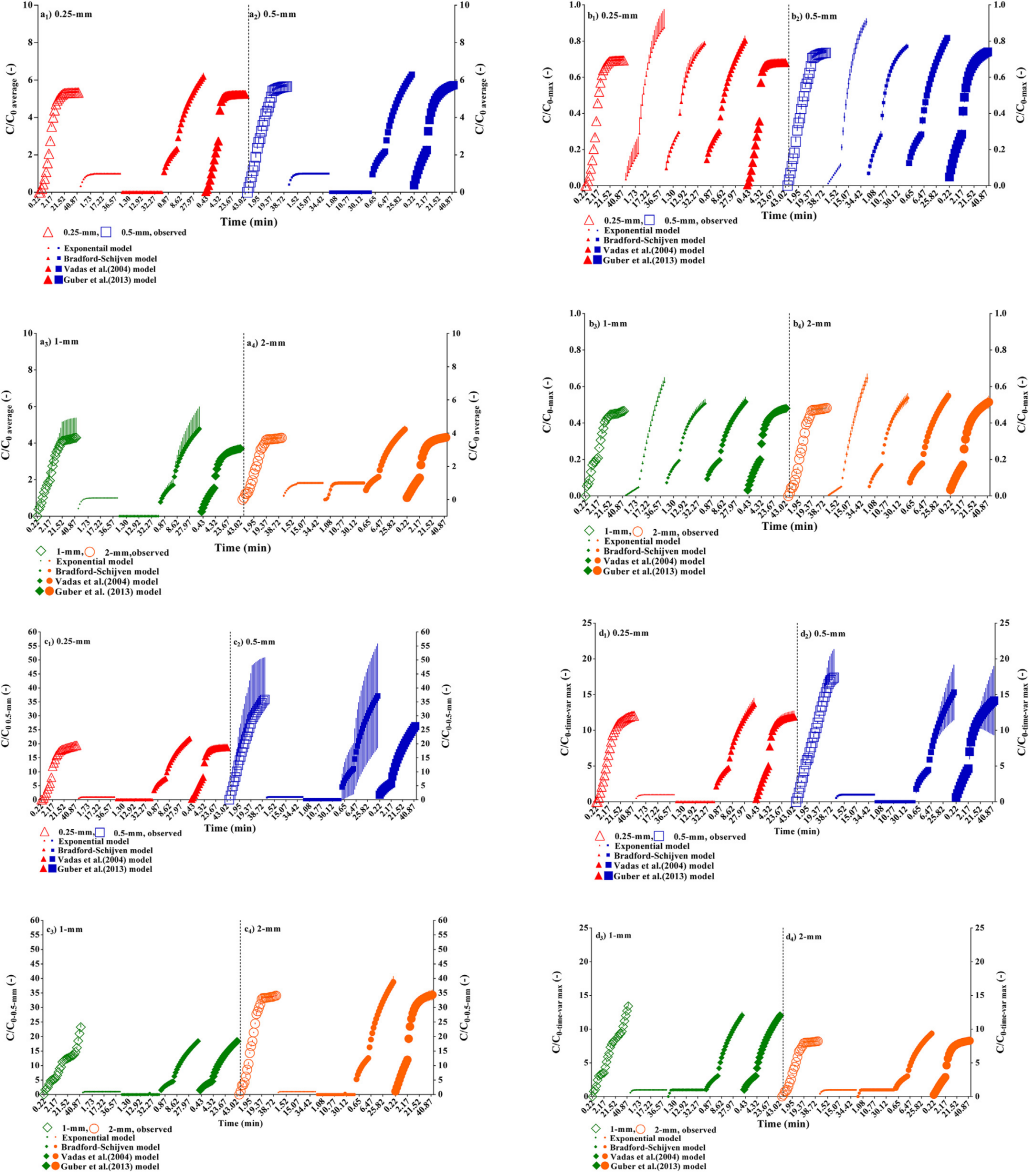
Modeling of the cumulative release bacteria curves from four different manure fraction sizes normalized using the (a_1_-_4_) average and (b_1_-_4_) maximum concentrations found in the air-dried bulk materials, as well as time variable concentration conditions including (c_1_-_4_) the concentration values of the 0.5-mm fraction, and (d_1_-_4_) maximum concentration values selected among all fractions at each pore volume (Time-var max), as the reference concentrations *C*_*0*_, during the leaching period. The bars represent standard deviation.

Four release curve models presented by Eqs. (1), (2), (3), and (4) were fitted to the experimental data using the target initial boundary conditions (Figure 4). The effect of manure size was significant for the three considered boundary conditions (*i.e.,* average, Max, and 0.5-mm fraction) (*p* < 0.0001), but not for time-variable boundary condition. This indicates the latter condition neutralized manure size effect and might not be a promising boundary condition to make CRCs normalize. The applied models estimated different values for a specific parameter (*e.g., k*_*e*_, *k*_*p*_*, β*, or *Er;* see Eqs. (1), (2), (3), and (4) concerning cell release or shape of CRCs (*p* < 0.05) with regard to the given boundary conditions for *C*_*0*_. The fitted parameters are presented in Tables 3a and 3b. The unreported values for parameters indicate unsuccessful simulation for the target model.

## 4 Discussion

### 4.1 Bacteria release curves

The decreasing and stationary modes (phases II and III) of release occupied a considerable time span of the release compared to the increasing mode (phase I) indicate a great mass of bacteria could easily release from the manure particles in short periods (*i.e.,* a more dynamic release below 1 PV, Figure 1), while cells could have long retardation in release. Bacteria in a suspension released from manure are distributed as individual cells, flocculated cells, cells attached to organic colloids, and particulates (McDaniel et al., 2013; Muirhead et al., 2005, 2006a, 2006b; Soupir et al., 2010). The observed cell release in tendency could be due to strong cell-particle binding (Liang et al., 2014, 2017; Zhao et al., 2014) and most probably to trapping cells in woody coarse materials and complex pore spaces of manure fractions (Blaustein et al., 2015; Schijven et al., 2004; Sepehrnia et al., 2017). Schijven et al. (2004) reported such release modes for parasites (e.g., *Cryptosporidium parvum oocysts* and *Giardia duodenalis cysts*) released from cow and cattle manures. They studied three combinations of calf and cow manures (calf manure, a 50% calf and 50% cow manure mixture, and a 10% and 90% cow manure mixture) and found that for a given manure type, the release efficiency of the small oocysts was higher (4–6 μm) than the larger cysts (8–12 μm). This was concluded that the cysts released from finer calf manure particles are more readily released than larger cow manure particles since water flows more easily through the larger textured cow manure. Pachepsky et al. (2009) indirectly described microbial release as the result of changes in the size distribution of suspended particles released from a dairy manure throughout rainfall. They observed that particle size distributions in manure runoff and leachate suspensions remained remarkably stable after 15 min of runoff initiation so that particles had the median diameter of 3.8 μm, and 90% of particles were between 0.6 and 17.8 μm. However, they did not trace the bacteria release and called for more information about the concurrent release of pathogens and manure particles during rainfall events. By extension, our results demonstrated that the finer (<0.25-mm) and larger (>1-mm) fractions of the studied manure control the intensity and the shape of cell release, respectively (Figure 1). We believe that the heterogeneity of manure fractionation from finer to larger (0.25-mm to 2-mm) increases the probability of fine particles and cell trapping as well as tailing and fluctuation in release due to the complex physics of pore spaces and the accessibility of the particles to flowing water within the pre spaces (Figure 1).

Multiple comparisons revealed that the PV effect on release concentration became trivial at the last PVs in stationary (phase III) (*e.g.,* at PVs of 5 and 6, 7 and 8, 7 and 9, or 8 and 9). This test also proved that the effect of manure size fractions was significant for the cell release in all cases (*e.g.,* 0.25-mm vs. 0.5-mm or 0.5-mm vs. 2-mm; *p <* 0.001) except for 0.25-mm vs. 2-mm which was approaching significance (*p* < 0.06), indicating that as the manure heterogeneity increased, a specific fraction may play a key role in the release of bacteria. The evidence is the similarity of shape and peak of the bacteria release from manure bulk to 0.25- and 2-mm fraction, respectively (Figure 1). This proportionally demonstrates the stronger effect of the fraction size on the bacteria release compared to PV (*e.g.,* volume/time of rainfall/leaching).

Some previous studies showed that the rate and extent of cell release vary among animal waste types and bacteria strain (Blaustein et al., 2015; Mawdsley et al., 1995; Soupir et al., 2010). *E. coli* was shown to be less often attached to soil particles than *enterococci* (Soupir et al., 2010). Blaustein et al. (2015) reported that physical properties of animal waste, especially the proportion of solids and liquids within the matrix, appear to strongly affect the dependency of release on total rainfall so that the relationship between microbial release and rainfall appears to be strongest for release from slurry, less for cowpats, and very weak for solid manure. Sepehrnia et al. (2017) concluded that dried poultry manure more readily released coliform when compared with cow and sheep manures counterparts. They indirectly attributed cell release to the manure particle size and concluded that poultry manure with a higher soluble mobile component could result in a higher free-cell bacteria transport, while, the greater amounts of wooden solid materials of cow and sheep manures acted as harbors and delineated a greater bacteria tailing in cell transport. In addition to the previous studies focused on cell release from soil particle fractionation (*e.g.,* Soupir and Mostaghimi, 2011; Guber et al., 2007b), our findings proved the importance of solid manure physics as a key role in cell release and fate.

### 4.2 Average and single maximum concentrations

The CRCs of manure fractions normalized using the average value (*C*_*0*_ = 10. 40 × 10^6^ CFU ml^−1^) and maximum concentration (*C*_*0*_ = 80.00 × 10^6^ CFU ml^−1^) of bacteria cells as the initial concentration are illustrated in Figure 2a, b, respectively. The original modes of the bacterial release, shown in Figure 1 are reflected in the normalized CRCs (Figure 2a) and compared to the results reported by Sepehrnia et al. (2014, 2017). However, although the single maximum concentration value (80.00 × 10^6^ CFU ml^−1^) was close to the previous influent of pure cell suspensions; *i.e.,* 100.0 × 10^6^ CFU ml^−1^ (Sepehrnia et al., 2014, 2017, 2018), the trend of the cell release was underestimated in the curves (Figure 2b), so that the maximum *C*/*C*_*0*_ was only 0.1 (Figure 1b). This indicates such a single influent concentration (*i.e.,* 100.00 × 10^6^ CFU ml^−1^) may not properly show the bacterial release trend from manures in the field systems. Thus, if solid manures are considered, the previous studies illustrate a great heterogeneity in reports (*i.e.,* CFU ml^−1^ or CFU g^−1^, etc.) rather than the normalized form (Mantha et al., 2017; Guber et al., 2005; Hruby et al., 2016) because, in most cases, determination of the exact initial concentration of bacteria is challenging (Guber et al., 2005; 2007a, b, 2013).

### 4.3 Time variable concentrations

The curves in Figure 2c show the release in different manure fractions normalized using time variable concentrations when the *C*_*0*_ were obtained from the 0.5-mm fraction, supposing that a specific fraction which had the greatest frequency of the highest concentration values controls the cell release from manure. Therefore, each PV concentration of the 0.5-mm fraction was considered as a *C*_*0*_. The results illustrate that there are many points in the release curves when the *C* were considerably higher than the *C*_*0*_. Furthermore, such time variable concentrations provided some unrealistic release trends either at the beginning (*e.g.,* 2-mm release curve) or end of the leaching (*e.g.,* 1-mm release curve) if the fractions are considered and compared with the original 0.5-mm release curve in Figure 1. We believe the 0.5-mm fraction as the *C*_*0*_ did not present information about the rate of bacteria decrease in the reference 0.5-mm fraction.

Figure 2d illustrates the normalized CRC using the time variable concentrations of the maximum values of each PV among different manure fractions. In comparison to Figure 2c, the changes of the *C*/*C*_*0*_ were promising because the values decreased and the maximum *C*/*C*_*0*_ values neared to 1. However, such variable concentrations did not show the trend of the bacteria release for the 0.5- and 1-mm fractions very well, particularly at the mid to end of the leaching period. The results were thus unrealistic if they are compared with the original release trend and previous reports (*e.g.,* Bradford et al., 2006; Sepehrnia et al., 2014, 2017, 2018).

In general, the CRCs from Figure 2a had a three-phase pattern in release and showed reasonably better peak values during leaching where they were separated for all different manure fractions similar to Figure 1. Therefore, considering a single value as the reference to achieve, the normalized CRC would be a promising and reasonable procedure using the average concentration of bacteria in all fractions.

### 4.4 Evaluation of the manure-treated soil BTCs

A large body of information has been reported in previous studies about bacteria transport and physical, chemical, and biological soil properties, and manure types (*e.g.,* Bradford et al., 2013; Engström et al., 2015; Unc et al., 2012, 2015). However, as mentioned earlier, this knowledge gap has still remained unexplored on how physics of solid manures impacts bacteria fate (Blaustein et al., 2015; Stocker et al., 2020a). This first needs information about how bacteria are released from solid manure matrix as addressed in 3.1 and 4.1. Another aspect is interpretation of the results for the solid-manure treated soils with regards to the possible initial boundary conditions for cell concentration. In this study, we showed that the average of bacteria concentrations during the 20 PV leaching period could be a promising index to produce normalized CRCs of manure release. However, it was necessary to find if the manure-treated soil BTCs could also be made to normalize using the given average. Therefore, all evaluations considered in Figure 2 were used to have dimensionless BTCs of a sandy manure-treated soil (Figure 3). Figure 3a, b show the BTCs of the soil using the variables in Figure 2 as the *C*_*0*_. The results highlighted that only the release trend of the cells from manures was reflected despite the soil, no matter which given reference concentration (*C*_*0*_; a single or time variable concentration) was evaluated. The manure-treated soil data was eventually assessed using the time variable concentration of each manure fraction for the corresponding PVs in the manure-amended soil effluent data (*i.e., C*/*C*_*0*_: 0.25 *C* and 0.25 *C*_*0*_, respectively). In other words, *C*_*0*_ in this case referred to the time variable bacteria concentration in which the PV was measured. The BTCs simultaneously showed the effects of both soil and manure. Several recent studies have illustrated the impacts of animal wastes and compost on bacteria transport (*e.g.,* Sharma and Reynnells, 2016; Sistani et al., 2010; Unc et al., 2012). Li et al. (2020) explored the occurrence and fate of human pathogenic bacteria in soil microcosms treated with two rates of swine, poultry or cattle manures and detected 30 pathogens in manure and soil samples. Of which, as revealed by co-occurrence pattern, *Pseudomonas syringae* pv. syringae B728a and *Escherichia coli* APEC O78 may deserve more attention because of their survival for a few days in manured soils and being possible hosts of antibiotic resistance genes. Thus, poultry manure had the highest level of pathogenic contamination, while, swine manure had a higher contribution to soil pathogenic communities than those from poultry or cattle manures in early days of incubation. Unc et al. (2012) confirmed the governing role of waste type on vadose-zone microsphere transport and concluded that retention is not necessarily facilitated by manure-microsphere-soil interactions but by manure-soil interactions. In addition to the previous studies, our data demonstrates that the cell release from manure is necessary to be also examined and evaluated in parallel to the properties of the bacterial contaminated soil and field systems.

### 4.5 Modeling and performance

All manure fraction leaching data showed a precipitous log-linear increase in the cumulative release at the beginning, which was followed by a much slower steady-state release mode for the rest of leaching (Figure 4). In general, two phases could be distinguished by slope shapes of each CRC for all considered boundary conditions (Figure 4), although a three-phase tendency can be traced for fractions 1-mm and 2-mm (Figure 4) as was proposed in the original data illustrated in Figure 1. This indicates a similar release rate for the phase II and III in the 0.25-mm and 0.5-mm fractions, illustrating the effects of physical manure heterogeneity on the bacterial release if compared with the larger fractions of 1-mm and 2-mm. Blaustein et al. (2015) reported two-stages for the cumulative release mode of *E. coli* from a synthetic manure mix (consisting of fresh cattle excreta combined with saw dust bedding) under different rainfall intensities. Hodgson et al. (2009) examined relative release kinetics of faecal indicator organisms from through a laboratory assay and found differences between *E. coli* and intestinal *enterococci* release originated from various manures. The order of *E. coli* release from the faecal matrices was dairy cattle slurry > beef cattle farm yard manure > beef-cattle faeces > sheep faeces. For intestinal *enterococci*, the magnitude order of release was dairy cattle slurry > beef-cattle faeces > beef cattle farm yard manure > sheep faeces. Weaver et al. (2016) evaluated *E. coli* and *Campylobacter* fate released from cowpats applied on soil lysimeters and found that the persistence of *E. coli* in the cowpats during the experiment is an important property which makes conditions more favorable for *E. coli* survival and growth. In corroboration with Weaver et al. (2016), but from the physical perspective, we believe for short-time leaching (*i.e.,* 43 min) the heterogeneity of solid manure controls persistence of *E. coli* release in tendency and makes lag-time following concentration-shock in release by supplying the second and third waves of the concentrations in the effluent shortly after beginning of the leaching. That can result in having the bimodal or multi-modal BTCs peaks for bacteria appeared in the effluent (*i.e.,* see 1-mm and 2-mm fractions in Figure 3e).

The estimated values for parameters were comparable to previous studies (*i.e*., Guber et al., 2013; Blaustein et al., 2015; Stocker et al., 2020a) that used a two-parametric- Bradford and Schijven (2002) model. Among the models studied, only the Vadas et al. (2004) model provided a unique value (0.3–0.4) for the dimensionless curve-shape parameter, *β*, regardless of the applied boundary conditions. The other reported parameters had high variability when either the models or the boundary conditions were compared. Stocker et al. (2020b) evaluated the removal of *Escherichia coli* and *enterococci* with runoff for two different manure consistencies (Liquid and solid dairy manure) and three manure weathering durations (one week and two weeks), and reported that Vadas et al. (2004) and Bradford– Shijven removal models performed similarly, while, the latter model was slightly more accurate, and the former model had better showed dependencies of parameter values on manure weathering. Notwithstanding, discussion of the parameters as a function of manure size needs to find the best boundary condition for the initial concentration as given in the following.

The performance of applied models was different based on the initial boundary conditions of bacteria concentration. Release kinetics appeared to be better simulated using all four models when the maximum boundary condition was considered (Figure 4b). The Bradford and Schijven (2002) model was very sensitive to the selected initial boundary conditions compared to the other models, so it was only fitted to the maximum boundary condition (Figure 4). The RMSE and AIC values for models are also presented in Tables 4a and 4b. The minimum values of the RMSE and AIC indices were found for the Max boundary conditions, indicating the best boundary condition to make release curves normalize (Table 4a). The unrealistic boundary conditions were the 0.5-mm fractions and Time-variable initial conditions because of the highest RMSE and the positive AIC values (see Table 4b). Bradford and Schijven (2002) and Guber et al. (2013) models had the lowest RMSE and AIC values, demonstrating better performance of these models to simulate release of bacteria cells than the exponential and Vadas et al. (2004) models. The Guber et al. (2013) model had slightly more negative AIC values than Bradford and Schijven (2002) model, indicating the best considered model for the present study (Table 3a). This illustrates that the model with greater number of fitting parameters increases the model flexibility and performance.

**Table 4a:** Average and standard deviation of RMSE and AIC parameters from the exponential, the Bradford and Schijven (2002), Vadas et al. (2004), and Guber et al. (2013). Models fitted to the effluent data under different manure fraction and boundary conditions (i.e., average and max values).

*C*_*0*_ = average	0.25-mm		0.5-mm		1-mm		2-mm	
model	RMSE	AIC	RMSE	AIC	RMSE	AIC	RMSE	AIC
Exponential	3.463 (±0.132)	73.151 (±2.206)	3.403 (±0.118)	72.140 (±2.010)	2.399 (±0.772)	50.053 (±17.467)	1.919 (±0.044)	38.927 (±1.332)
Bradford and Schijven (2002)	–	–	–	–	–	–	1.988 (±0.112)	43.248 (±3.234)
Vadas et al. (2004)	0.832 (±0.019)	-7.227 (±1.309)	0.500 (±0.032)	-36.783 (±3.727)	0.367 (±0.086)	-55.734 (±13.196)	0.403 (±0.017)	-49.309 (±2.423)
Guber et al. (2013)	0.239 (±0.022)	-77.243 (±5.326)	0.297 (±0.043)	-64.777 (±8.379)	0.114 (±0.033)	-121.390 (±17.151)	0.161 (±0.015)	-100.190 (±5.732)
*C*_*0*_ = max	0.25-mm		0.5-mm		1-mm		2-mm	
model	RMSE	AIC	RMSE	AIC	RMSE	AIC	RMSE	AIC
Exponential	0.164 (±0.004)	-103.829 (±1.311)	0.129 (±0.009)	-117.924 (±3.926)	0.119 (±0.002)	-122.448 (±1.199)	0.110 (±0.003)	-127.149 (±1.824)
Bradford and Schijven (2002)	0.079 (±0.003)	-143.759 (±1.861)	0.042 (±0.004)	-180.849 (±5.066)	0.031 (±0.003)	-197.787 (±4.815)	0.041 (±0.001)	-181.670 (±0.899)
Vadas et al. (2004)	0.108 (±0.002)	-125.560 (±1.309)	0.065 (±0.004)	-155.116 (±3.727)	0.040 (±0.003)	-183.201 (±4.803)	0.052 (±0.002)	-167.641 (±2.423)
Guber et al. (2013)	0.032 (±0.004)	-193.954 (±6.40)	0.038 (±0.006)	-184.576 (±8.379)	0.015 (±0.003)	-237.714 (±12.617)	0.021 (±0.002)	-218.523 (±5.732)

**Table 4b:** Average and standard deviation of RMSE and AIC parameters from the exponential, the Bradford and Schijven (2002), Vadas et al. (2004), and Guber et al. (2013) Models fitted to the effluent data under different manure fraction and boundary conditions (i.e., 0.5-mm and Time-variable max values).

*C*_*0*_ = 0.5-mm	0.25-mm		0.5-mm		1.0-mm		2.0-mm	
Model	RMSE	AIC	RMSE	AIC	RMSE	AIC	RMSE	AIC
Exponential	14.174 (±0.462)	153.188 (±1.884)	23.518 (±17.090)	178.148 (±38.595)	10.827 (±0.447)	139.253 (±2.406)	25.224 (±0.968)	188.314 (±2.215)
Bradford and Schijven (2002)	–	–	–	–	–	–	–	–
Vadas et al. (2004)	2.608 (±0.075)	57.650 (±1.651)	2.290 (±2.635)	35.596 (±62.510)	1.552 (±0.199)	28.629 (±7.485)	3.698 (±0.155)	79.257 (±2.423)
Guber et al. (2013)	0.974 (±0.053)	2.365 (±3.083)	1.055 (±0.022)	9.055 (±1.234)	1.554 (±0.199)	31.208 (±7.478)	1.477 (±0.142)	28.375 (±5.732)
*C*_*0*_ = Time-var max	0.25-mm		0.5-mm		1.0-mm		2.0-mm	
Model	RMSE	AIC	RMSE	AIC	RMSE	AIC	RMSE	AIC
Exponential	8.359 (±0.399)	124.242 (±2.734)	9.151 (±1.770)	128.855 (±10.715)	6.803 (±0.264)	112.309 (±2.267)	5.423 (±0.240)	99.147 (±2.550)
Bradford and Schijven (2002)	–	–	–	–	6.875	115.260	–	–
Vadas et al. (2004)	1.365 (±0.039)	21.483 (±1.655)	1.176 (±0.311)	11.288 (±16.880)	0.631 (±0.059)	-23.453 (±5.625)	0.892 (±0.037)	-3.243 (±2.423)
Guber et al. (2013)	0.552 (±0.057)	-28.703 (±5.816)	0.621 (±0.174)	-23.132 (±15.365)	0.631 (±0.062)	-20.960 (±5.837)	0.356 (±0.034)	-54.124 (±5.732)

The parameters values using the Max boundary condition can be considered as the realistic dataset (Table 3a). For the exponential model (Eq. (1)); the *k*_*e*_ values had a slight decrease in tendency as the manure particle size increased. The data were three-fold smaller for the 0.25-mm and 0.5-mm fractions, and six-fold for the 1-mm and 2-mm fractions, than the values reported by Blaustein et al. (2015) for *E. coli*. The *k*_*p*_ remained constant in the range of 0.0157–0.0234, while *β* values in the Bradford and Schijven (2002) model showed slight increase from 1.372 to 2.073 as the manure fraction size increased (Tables 3a and 3b). Blaustein et al. (2015) also reported similar values for the *β*, with higher standard deviation (1.9785 ± 2.0230) for the *E. coli* using the Bradford and Schijven (2002) model compared to our results. The parameter *k*_*p*_ in the Vadas et al. (2004) model showed a slight decrease as the manure particle size increased, but the *β* values remained constant. The results for exponential and Vadas et al. (2004) models (Tables 3a and 3b) were comparable with Stocker et al., 2020a, Stocker et al., 2020b data for *E. coli* released from a dairy solid manure with 25% consistency after two weeks weathering. The values for parameters *k*_*p*_, *β*, *Er* using the Guber et al. (2013) model were not substantially changed between manure fractions, however, the minimum values for *k*_*p*_ and *β* were found for the smallest fraction (*i.e.,* 0.25-mm) (Tables 3a and 3b). The value of *Er* was between 0.51 to 0.83, which was also close to the value (0.551 ± 0.121) reported for *E. coli* by Guber et al. (2013).

## 5 Conclusions

The CRCs are reasonably normalized using an average bacteria concentration found in all manure fractions. However, the soil BTCs treated with the manure fractions could be reasonably interpretable for transport, retention, and release processes when the time variable concentration of bacteria released from manure was considered as the *C_0_* for the corresponding soil effluent concentration (*C*) for each fraction.

The model simulation showed that and Guber et al. (2013) models provided reasonable parameter information for cell release when a single maximum concentration was considered as the initial influent concentration. Therefore, our findings revealed that physics of solid manures controls cell release; both the bacterial release curves from manures and the bacteria BTCs of manure-treated soils should be thus separately evaluated for manure management practices in fields. With this study, we propose modification of solid cow manure bulk, as a management protocol, to fractions smaller than 0.5-mm and 1-mm to minimize bacterial contamination risk. Finally, we suggest extending this study to assess the bacterial release from different solid manures (*e.g.,* poultry, sheep, calf, and cattle) applied to agricultural fields.

## Declarations

### Author contribution statement

Nasrollah Sepehrnia: Conceived and designed the experiments; Analyzed and interpreted the data; Contributed reagents, materials, analysis tools or data; Wrote the paper.

Sayyed-Hassan Tabatabaei: Conceived and designed the experiments; Wrote the paper.

Hamdollah Norouzi: Performed the experiments.

Mohsen Gorakifard: Contributed reagents, materials, analysis tools or data; Wrote the paper.

Hossein Shirani; Fereidoun Rezanezhad: Analyzed and interpreted the data; Wrote the paper.

### Funding statement

This work was supported by Shahrekord University. Nasrollah Sepehrnia was supported by Alexander von Humboldt Foundation and Postdoctoral Fellowship at Leibniz University of Hannover, Germany.

### Data availability statement

Data will be made available on request.

### Declaration of interests statement

The authors declare no conflict of interest.

### Additional information

No additional information is available for this paper.
